# Microvolume DNA extraction methods for microscale amplicon and metagenomic studies

**DOI:** 10.1038/s43705-021-00079-z

**Published:** 2021-12-17

**Authors:** Anna R. Bramucci, Amaranta Focardi, Christian Rinke, Philip Hugenholtz, Gene W. Tyson, Justin R. Seymour, Jean-Baptiste Raina

**Affiliations:** 1 Climate Change Cluster (C3), University of Technology Sydney, Sydney, NSW 2007 Australia; 2 Australian Centre for Ecogenomics, School of Chemistry and Molecular Biosciences, The University of Queensland, Brisbane, QLD 4072 Australia; 3 Centre for Microbiome Research, School of Biomedical Sciences, Queensland University of Technology, Translational Research Institute, Brisbane, QLD 4102 Australia

**Keywords:** Water microbiology, Next-generation sequencing, Metagenomics

## Abstract

Investigating the composition and metabolic capacity of aquatic microbial assemblages usually requires the filtration of multi-litre samples, which are up to 1 million-fold larger than the microenvironments within which microbes are predicted to be spatially organised. To determine if community profiles can be reliably generated from microlitre volumes, we sampled seawater at a coastal and an oceanic site, filtered and homogenised them, and extracted DNA from bulk samples (2 L) and microvolumes (100, 10 and 1 μL) using two new approaches. These microvolume DNA extraction methods involve either physical or chemical lysis (through pH/thermal shock and lytic enzymes/surfactants, respectively), directly followed by the capture of DNA on magnetic beads. Downstream analysis of extracted DNA using both amplicon sequencing and metagenomics, revealed strong correlation with standard large volume approaches, demonstrating the fidelity of taxonomic and functional profiles of microbial communities in as little as 1 μL of seawater. This volume is six orders of magnitude smaller than most standard operating procedures for marine metagenomics, which will allow precise sampling of the heterogenous landscape that microbes inhabit.

Interest in extraction methods capable of recovering ‘trace’ or low-input amounts of DNA has grown rapidly in a wide range of research fields, including forensics [1], the human microbiome [2], soil microbiology [3], ancient DNA [4–6] and aerosol research [7]. For instance, recent studies successfully characterised microbial consortia from 1 mg of soil, when standard approaches typically recommend using 250 times this amount [8]. In contrast, the aquatic microbial ecology field is lacking low-input DNA extraction techniques. Currently, the recovery of microbial DNA from aquatic environments typically involves the filtration of large volumes (2–200 L) of seawater [9–11]. This large volume sampling means that targeted characterisation of microbial communities inhabiting microenvironments (e.g. surrounding marine protists, attached to marine snow particles, or within-host cavities) is not possible and spatial heterogeneity in aquatic bacterial community composition and abundance is therefore often ignored and averaged out [12, 13], resulting in the potential misinterpretation of important ecological patterns [14–17]. Within this context, the critical bottleneck has been the capacity to obtain sufficient amounts of quality DNA from small volume samples (i.e. microliters) for downstream analysis (e.g. amplicon sequencing and metagenomics) [18]. Here we present and validate two novel microvolume DNA extraction techniques that employ either i) physical or ii) chemical lysis to rupture cells (Fig. 1a), facilitating precise assessment of the taxonomic composition and functional potential of microbial assemblages from samples as small as 1 µL.

**Fig. 1:**
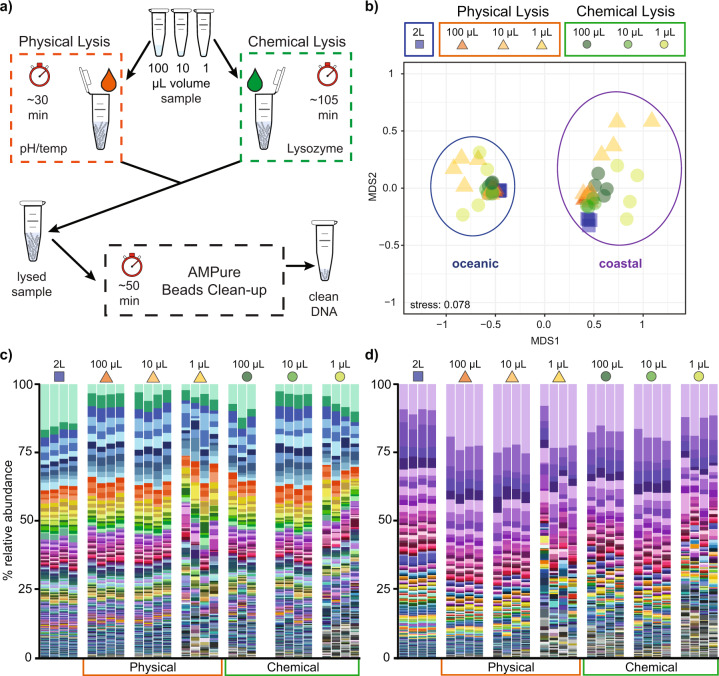
Microvolume DNA extraction method and resulting taxonomic composition of 16S rRNA gene amplicons from the different extraction methods and seawater volumes tested. **a** Microvolume (e.g. 100 µL, 10 µL, 1 µL) DNA extraction protocol for both physical lysis (which relies on both pH and thermal shock to lyse cells and takes ~30 min for a batch of ten samples) and chemical lysis (which uses lysozyme and SDS to lyse cells and takes ~105 min for a batch of ten samples). Cell lysate is then cleaned using AMPure beads, which takes about 50 min and results in purified DNA ready to be used in a variety of downstream applications (e.g. amplicon sequencing, shotgun metagenomics, Nano-string, qPCR, etc). A more detailed version of this schematic is presented in Fig. S1. **b** Non-metric multidimensional scaling (nMDS) plot based on Bray–Curtis distance between samples. Ovals highlight statistically different consortia between the two sampling sites (PERMANOVA, *p* < 0.001, Table S8); within those ovals the consortia were not statistically different irrespective of volume or extraction method (PERMANOVA, *p* > 0.4, Tables S9, S10). Microbial diversity of amplicon sequence variants (ASVs) (>0.1% relative abundance in at least one replicate extraction) are displayed for each site separately: **c** oceanic sampling site and **d** coastal sampling site. ASVs are ordered by decreasing relative abundance. ASVs that were rare in the bulk 2 L extraction (<0.1% relative abundance), but made up >0.1% of one of the microvolume extractions, are coloured in various shades of blue in all extractions. ASVs absent from the 2 L extraction are coloured in various shades of grey and present at the bottom of the bar chart. The full legend for **c** and **d** can be found in Tables S11, S12. Note: the colour palettes used for **c** and **d** are different.

Both microvolume DNA extraction techniques were tested on samples collected from two marine locations: an oceanic (34°07′06′′S 151°13′09′′E) and a coastal (33°54′52.9′′S 151°16′04.5′′E) site near Sydney (Australia), enabling comparison of environments harbouring distinct assemblages, low microbial abundances, and varying water chemistry. Twenty litres of surface seawater collected from each site were first gently poured through a 165-µm mesh to remove particulates and then homogenised by mixing in a carboy to reduce microscale heterogeneity. DNA was then extracted from different volumes in quadruplicate: (i) 2 L were filtered and extracted with a commonly used DNA extraction kit (DNeasy PowerWater, QIAGEN), which acted as a benchmark; (ii) 100 µL, 10 µL, and 1 µL were extracted using our two microvolume extraction approaches (Fig. 1a, Table S1). This volume range was used to assess the ability of each method to characterise microbial communities at scales that are more relevant to the ecology of the microorganisms. For each microvolume sample, cells were lysed by exposure to either (i) an alkaline solution (including KOH and dithiothreitol to cleave protein disulphide bonds [19]) followed by a thermal shock (physical lysis), or (ii) a mixture of broad spectrum lytic enzymes and surfactants (Lysozyme—SDS—proteinase K; chemical lysis) (Fig. S1). Free DNA was then captured using magnetic beads commonly used for library preparation [20–22], before being purified and eluted (Tables S2-S3). To maximise DNA recovery, the entire extraction protocol was carried out in a single tube per sample. Multiple negative controls were collected alongside the seawater samples to identify and remove contaminants. Extracted samples were then characterised via amplicon sequencing (16S rRNA gene, Tables S4–S6) and those from the oceanic site were further explored using shotgun metagenomics.

All microvolume DNA extractions, including the 1 µL samples, were successfully amplified and sequenced. Their taxonomic composition was compared to the 2 L samples after stringent quality filtration and contaminant removal (Fig. S2). The proportion of reads identified as contaminants increased as extracted volume decreased, ranging from an average of 0.12% (100 µL samples) to 4.5% (1 µL samples) for the physical lysis, and from 6.99% (100 µL) to 54.5% (1 µL) for the chemical lysis (Table S7). Despite the large proportion of reads lost due to contaminants in some of the chemical lysis samples, taxonomic profiles from all DNA extraction methods and volumes were statistically indistinguishable within each site (PERMANOVA, *p* > 0.4; Fig. 1, Figs. S3, S4, Tables S8–S12). In addition, the amplicon sequence variant (ASV) proportions derived from the microvolumes were highly correlated to the standard large volume approach (Spearman’s correlation, *p* < 0.0001; Figs. S5–S7), highlighting a high level of fidelity between the microvolumes and the 2 L samples. These findings demonstrate that both microvolume extraction techniques enable reliable characterisation of microbial community composition down to 1 µL of seawater.

To determine if consistent and representative functional profiles could also be derived from microvolume extractions, samples from the oceanic site were sequenced using shotgun metagenomics and functional and taxonomic profiles were compared to those generated using the standard 2 L extraction (with 20 cycles of amplification used for the microvolume samples based on previous optimisations [21]). Each of the microvolume extractions resulted in an insertion size range that was within the recommended length for the Illumina protocol (200–340 bp; Figs. S8, S9, Table S13). Despite larger proportion of contaminants identified in the chemical lysis approach, this extraction outperformed the physical lysis method for the 1 µL samples, with an average of nine times more contigs assembled (Fig. S8, Table S13). Additionally, the 1 μL physical lysis extractions yielded smaller library sizes with fewer unique reads, did not assemble as well (producing on average 51.5 times fewer contigs longer than 500 bp than the 1 µL chemical lysis), and exhibited large deviations from the other metagenomes (Figs. S8–S10, Table S13). Irrespective of extraction or volume, the assigned functional profiles of the microbial assemblages were not statistically different (PERMANOVA, *p* > 0.05; Fig. 2, Figs. S11, S12, Tables S14, S16). These results confirm that the microvolume extractions accurately and reproducibly capture the functional capacity of the marine microbial consortia and that microscale sampling schemes can be used to characterise microbial metabolic capacities from 1 µL of seawater (Figs. 2,  S13).

**Fig. 2:**
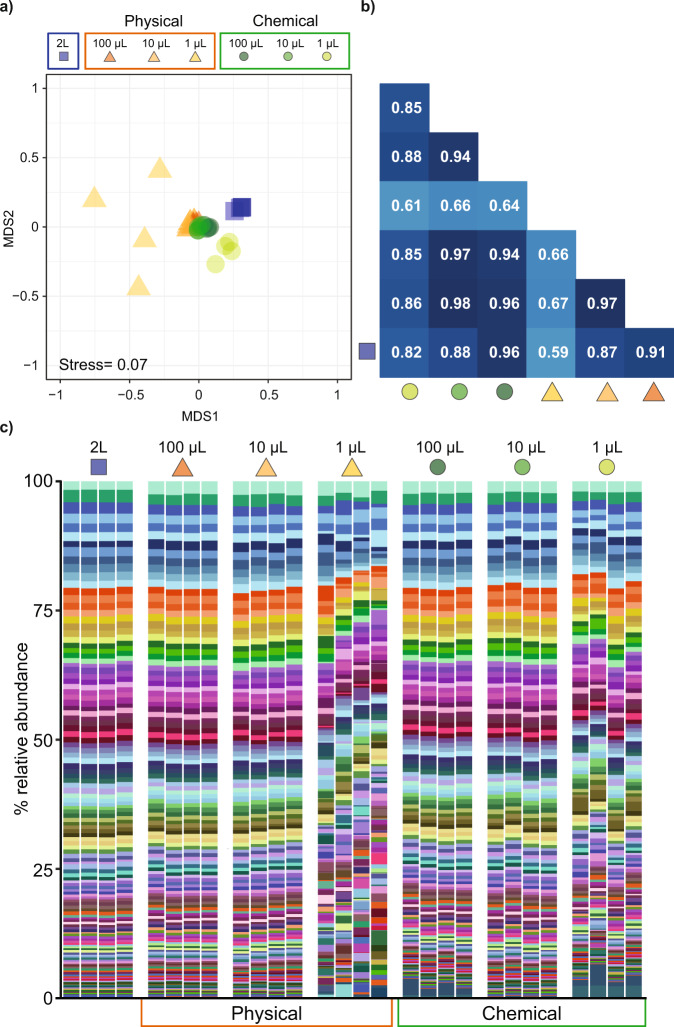
Functional composition of the seawater metagenomes from the oceanic site using the different methods and seawater volumes. **a** Non-metric multidimensional scaling (nMDS) plot based on Bray-Curtis distance between the annotated genes from prokaryotes. All samples were statistically indistinguishable (PERMANOVA *p* > 0.05, Table S14). **b** Spearman’s correlation analysis of the KO-based averaged functional profile of the annotated genes with at least 100 reads, all correlations shown are statistically significant (*p* < 0.0001). **c** Metagenomic genes (>0.3% relative abundance in at least one sample) identified from the 2 L extraction; physical lysis (100 µL, 10 µL, 1 µL); and chemical extractions (100 µL, 10 µL, 1 µL). The full legend for **c** can be found in Table S16.

We were able to co-assemble metagenome assembled genomes (MAGs; completion: >50%, redundancy: <20%) from the microvolume extractions. Specifically, we recovered: 19 MAGs from the 100 µL chemical lysis, 6 MAGs from the 10 µL chemical lysis, and 7 MAGs from the 100 µL physical lysis (in comparison, we recovered 20 MAGs from the 2 L; Table S17). We identified 6 MAGs in common between the 2 L extractions and the 100 µL chemical lysis, while the 10 µL chemical lysis and 100 µL physical lysis both shared 3 MAGs with the 2 L extractions (Table S18). Although it was not possible to assemble MAGs from the 1 µL samples (probably due to their lower numbers of contigs larger than 500 bp; Fig. S8), we anticipate that an increased number of replicates from this microvolume will enable their assembly.

Both microvolume extractions are (i) more cost effective than standard large volume extractions (e.g. PowerWater ($10.38 USD/sample)), costing $7.09 USD/sample when extracting 100 µL and only $0.72 USD/sample when extracting 10 µL or less; and (ii) much faster than standard extractions, given that filtration of the samples is not required (PowerWater extraction takes ~120 min, plus 30 to 120 min per sample to filter the required volume of water). Based on the results of our benchmarking tests, we suggest that the physical lysis extraction protocol is the most suitable for sample volumes greater than 10 µL, it is faster (taking ~80 min/batch of ten samples), and produces data with high levels of fidelity compared to standard approaches. However, the chemical lysis extraction is better suited for the smallest volume tested here (1 µL), enabling accurate characterisation of microbial communities with both amplicon sequencing and shotgun metagenomics. At this small volume, the variability between replicates increased significantly compared to 2 L for both amplicon sequencing (Figs. S14–S16, Tables S19–S27), and metagenomes (Figs. S10, S13, Table S28), which is consistent with prediction of heterogeneity at the microscale and indicates that despite prefiltration and homogenisation, some heterogeneity persisted in our samples.

Here we introduce and validate two DNA extraction approaches that facilitate reliable examination of both the taxonomic composition and metabolic potential of microbial communities in as little as 1 µL of seawater. Further reduction of contaminants originating from the chemical lysis reagents (as outlined in the methods) may allow successful extraction of nanolitre samples. In addition, these microvolume extractions enable the characterisation of microbial communities without the biases induced by filtration, which might be advantageous when studying viruses. We therefore believe that the DNA extraction methods presented here have the potential to change how we study aquatic microbial ecology, enabling researchers to assess the distributions and ecological interactions of microorganisms at the minute scales that are often more relevant to their ecology.

## Supplementary information

Supplementary Information

Supplementary Tables
