# Supplementary material for: Microvolume DNA extraction methods for microscale amplicon and metagenomic studies
Source: ISME Commun. 2021 Dec 17;1:79. doi: 10.1038/s43705-021-00079-z (PMC9723667; doi:10.1038/s43705-021-00079-z)
Supplement: Supplementary file 1 — Supplementary Information [file 43705_2021_79_MOESM1_ESM.docx]

**Supporting Information**

**Microvolume DNA Extraction Methods for Microscale Amplicon and Metagenomic Studies**

Anna R. Bramucci^1^, Amaranta Focardi^1^, Christian Rinke^2^, Philip Hugenholtz^2^, Gene W. Tyson^3^, Justin R. Seymour^1^ and Jean-Baptiste Raina^1^

^1^﻿Climate Change Cluster (C3), University of Technology Sydney, Sydney, NSW 2007, Australia

^2^ Australian Centre for Ecogenomics, School of Chemistry and Molecular Biosciences, The University of Queensland, Brisbane, QLD 4072, Australia

^3^ Centre for Microbiome Research, School of Biomedical Sciences, Queensland University of Technology, Translational Research Institute, Brisbane, QLD 4102, Australia

**Methods**

Sampling

Seawater samples were collected at a coastal site, Clovelly Bay (33°54'52.9"S 151°16'04.5"E), as well as at an oceanic site, Port Hacking (34°07'06"S 151°13'09"E) in October 2019. Surface seawater was pre-filtered through a 165 µm nylon mesh (Sefar Nitex, Sefar Inc., Switzerland) on site, transported to the laboratory (Clovelly Bay: <30 min, Port Hacking: < 3 hrs), and then homogenized into a 20 L carboy prior to filtration. The 20 L carboy was swirled occasionally while filtering 2 L onto replicate membrane filters (Millipore, USA, 0.22 µm; n=4), which were stored at -80°C until extracted (< 1 month). Simultaneously, 1 mL replicates of seawater were pipetted from the 20 L carboy into cryotubes (n=8), flash frozen in liquid nitrogen, and stored at -80°C until extracted (< 1 month).

Flow cytometry

Bacterial cell abundance of the oceanic site (Port Hacking) and the coastal site (Clovelly) was enumerated immediately after sampling using flow cytometry (Beckman Coulter Cytoflex LX, Indianapolis, USA). Samples were preserved with 2% glutaraldehyde, stained with SYBR green 10,000X (1:10,000 dilution; Invitrogen, USA), and incubated in the dark for 15 min before analysis. Filtered MilliQ water was used as sheath fluid. For each sample, forward scatter (FSC), side scatter (SSC), and green (SYBR) fluorescence were recorded. The samples were analysed at a flow rate of 25 μL min^−1^. Microbial populations were characterized according to SSC and SYBR Green fluorescence and cell abundances were calculated by running a standardized volume of sample (50 µl). The number of cells quantified from the bulk seawater was used to calculate the cell densities present in each extracted volume (Table S1).

Large-volume DNA Extractions:

Four replicate filters were extracted using a widely used PowerWater DNA extraction kit (DNeasy PowerWater Kit, QIAGEN, Germany) according to the manufacturer's instructions. DNA was eluted in 30 µL elution buffer. Three replicates of blank PowerWater DNA extractions were performed using kit reagents to identify contaminants.

Microvolume DNA Extractions:

1) Microvolume Sample Preparation:

All tubes used during this protocol were crosslinked in a UV crosslinker (CX-2000 UV Cross-linker 8-watt 254nm UV; Upland, CA, USA) on maximum energy for 1 hr prior to use. All buffers and MilliQ water used (except for the Lysozyme, Proteinase-K and SDS found in the Lysozyme extraction technique) were crosslinked on ice for 1 hr prior to use to further minimize the influence of potential contaminants in the microvolume extractions (1). The entire procedure for both microvolume DNA extraction techniques was performed in a DNA/RNA UV-cleaner box UVC/T-AR (BioSan, Latvia). We advise prolonged crosslinking of dry materials and cross-linking liquids for up to 2 hr on ice to further eliminate potential contaminants.

Four replicate 1 mL samples were thawed, homogenized, and aliquoted into duplicate crosslinked tubes of each of the following volumes: 100 µL, 10 µL, and 1 µL, for extraction using either the physical lysis or chemical lysis DNA extraction technique (Figure S1).

2a) Physical Lysis (*a step-by-step protocol is also available at the end of the supplementary information*)

Lysis buffer was added to each microvolume sample (according to the volumes in Table S2), followed by inversion and quick spin. The samples were then incubated at room temperature for 10 min followed by an incubation at -80°C for at least 10 min (samples can stay in the freezer for up to 4 hr). Samples were then flash thawed on a heat block (55°C) until completely liquid (<5 min). As soon as the samples were thawed, 75 µL of crosslinked STOP buffer (according to the volumes in Table S2) were added and homogenised immediately by pipetting up and down vigorously (followed by tube inversion and quick spin). Note: do not incubate samples for longer than indicated as DNA can be damaged by the alkaline nature of the lysis buffer. Following lysis, samples were then directly processed to the next step (see AMPure beads DNA clean-up below).

2b) Chemical Lysis (*a step-by-step protocol is also available at the end of the supplementary information*)

Lysis buffer was added to each microvolume sample (according to the volumes in Table S3), followed by inversion and quick spin. Lysozyme (100 mg/mL) in crosslinked MilliQ (according to the volumes in Table S3) was added to each sample, followed by inversion and quick spin. The samples were incubated at 37°C for 45 min. Sodium dodecyl sulfate (SDS; 25%) in crosslinked MilliQ (according to the volumes in Table S3) was added to the samples, and tubes were incubated at 70°C for 10 min on a heat block. Samples were then allowed to cool completely to room temperature, then the correct volume of proteinase K (20 mg/mL; see Table S3) was added, followed by inversion and quick spin. The samples were then incubated at 37°C for 45 min and then directly processed to next step (see AMPure beads DNA clean-up below).

3) AMPure beads DNA clean-up (*a step-by-step protocol is also available at the end of the supplementary information*)

AMPure XP beads (Beckman Coulter Inc., USA) were added into each lysed sample (volume according to Tables S2-S3), mixed by inversion, followed by a quick spin, then incubate for 15 min at room temperature. The sample containing beads were placed on a magnetic stand for 10 min or until liquid becomes clear (beads will be pulled by the magnet, leaving the supernatant clear). While keeping the tubes securely in the magnetic rack, the supernatant was removed and discarded with a pipette, being careful not to disrupt the beads from the magnet. The beads were washed twice with 80% ethanol (pure EtOH, diluted to 80% directly before use using crosslinked MilliQ) (see volumes in Tables S2-S3) or until beads are completely submerged (additional EtOH might be required for larger extraction volumes). Note: if the beads are disrupted or dislodged during cleaning, eject the beads back into the tube and allow the tube to remain on the magnetic stand until the liquid is once again clear, before returning to the washing procedure.

After the second washing step, residual EtOH was removed using a 20 µL pipette (without disrupting the beads) and tubes were left to air dry on the magnetic stand for 15 min. Any remaining EtOH droplets were carefully pipetted out before removing the tubes from the magnetic stand and adding 10-20 µL of elution buffer (10 mM Tris-HCl). Beads were carefully resuspended in the elution buffer by pipetting up and down a set number of times (e.g., 5-10, depending on the volume of beads to be resuspended). After an incubation at room temperature for 2 min, tubes were placed back on the magnetic stand until the liquid cleared (2-10 min). As much as possible of the clear elution buffer (now containing DNA) was transferred to a new tube to use as input for sequencing. Purified DNA was stored at -80°C until required, given the small amounts of DNA it is advised to rapidly (< 6 months) use it for downstream applications.

Expected time for the DNA clean-up of one sample is approximately 50 min for 10 samples. Finally, it is also possible to do only one EtOH wash step for the physical lysis protocol (two washing steps are strongly recommended for the chemical lysis protocol to ensure all EDTA is removed). Additionally, this DNA clean-up protocol is compatible with liquid handling robots and can be carried out using magnetic well plates.

**Amplicon Sequencing**

To characterise bacterial community composition in the different sample volumes, the 16S rRNA V3-V4 region was amplified using primers 341F and 805R primers (Table S4) (2). Details of the master mix for all different volumes is presented in Table S5. PCR was performed in the following way for all samples: 95°C for 3 min, followed by 25 cycles of: [95°C (30 s); 55°C (30 s); 72°C (30 sec)], and finally 72°C for 5 min, then hold at 4°C until PCR clean-up.

PCR clean-up was performed using AMPure XP beads (Beckman Coulter Inc., USA) to purify PCR products from primers, without changes to the manufacturer's protocol. The input of cleaned PCR-product into the indexing PCRs and sequencing reaction differed based on the extraction volumes (Tables S5-S6) and the protocol was adjusted to 10 rounds of amplification. The PCRs were cleaned again using AMP XP beads and normalized using the SequalPrep Normalization Plate Kit (Thermo Fischer, USA) to manufacturer’s specifications, and eluted into 20 µL. After normalization, 4 µL of the 2 L extraction and 10 µL of all microvolume extractions were pooled. Then 120 µL of the pooled amplicons underwent a left side bead clean-up and were eluted into 30 µL. Cleaned pooled amplicons were then sequenced on a MiSeq platform (2 × 300bp) at the Ramaciotti Centre for Genomics (UNSW, Sydney, NSW).

Amplicon sequencing processing

Amplicon read processing, statistical analysis, and figures were done in *R* (3). Bacterial 16S rRNA gene paired end Illumina R1 and R2 reads were processed using the DADA2 pipeline (4). Reads with any ‘N’ bases were removed and bacterial V3-V4 primers were truncated using Cutadapt (5). R1 and R2 were trimmed to remove low quality terminal ends (R1= 260; R2= 250), to produce the highest number of merged reads after learning error rate and removing chimera sequences (77.92% of raw reads produced high quality merged sequences). Amplicon sequence variant (ASV) table containing high quality unique ASVs was then annotated using 50% probability cut-off SILVA nr_v138 classifier (https://www.arb-silva.de/fileadmin/silva_databases/release_138/Exports/SILVA_138_SSURef_tax_silva.fasta.gz, DOI 10.5281/zenodo.3731174).

Quality filtration and removal of Contaminants

The quality ASV table was secondarily filtered to remove ASVs not annotated to kingdom Bacteria, as well as any annotated as mitochondria. Contamination is an important aspect of working with microvolume extractions of DNA for both amplicon and metagenomic sequencing. While contaminants are often present in DNA extraction kits, their contribution to the resulting sequences is often greater when dealing with very low DNA input extractions (6). For this reason, it is critical to extract and sequence blank extractions for the various extraction techniques used. Here we extracted and sequenced 2 extraction blanks for each of the 3 extraction types tested, as well as one PCR sequencing blank (which contained PCR reagents used on all of the samples). Each blank was treated identically to the samples (extracted using the same method). The ASV table was then secondarily filtered: all ASVs that were identified within the PCR blank were removed from all of the samples (if present). Then ASVs representing >0.03% of each extraction blank were eliminated from the appropriate extraction type (i.e., chemical lysis, physical lysis, or PowerWater). Using this stringent approach to remove contamination, we found that most of the contaminants had a very low relative abundance, with only 13 ASVs at an abundance greater than 1% in any sample (and an additional 10 ASVs at an abundance greater than 0.1% in any sample). The notable exception was the 1 µL chemical lysis samples, which contained a higher relative proportion of contaminants, the most abundant belonging to the genera *Shewanella* (18.3-23.7%), *Pantoea* (10.2-21%), *Pseudomonas* (7.3-16.7%), and *Marinomonas* which made up to 1% of one sample at the Oceanic site. One replicate of the 100 µL chemical lysis failed to produce any usable reads and was therefore not included in our analysis.

Rarefying ASVs to account for uneven sequencing depth

The microbial diversity across all of the low input extractions was highly similar across all replicates and treatments even when rarefying at different depths (Figure S3), however due to differences of sample input into the sequencing reaction (7,8), the total reads per sample were variable between the extraction volumes (ranging from 5,727 to 341,281 reads/sample) (Figure S2). The samples with the most variable and lowest number of reads all belonged to the 1 µL physical lysis extraction (Figure S2). Data were rarefied at a range of depths using vegan ‘*rrarefy*’ (from 5,500 to 30,000 reads), the effect of these different depths ranged from full inclusion to full exclusion of the 1 µL physical lysis extraction samples (Figure S3). To include all sample volumes, a rarefaction to 5,500 reads was used for the remainder of this work. After rarefying, singletons were removed, and the clean file was split into a bacterial file (chloroplasts removed) and a chloroplast file (bacterial reads removed). Relative abundances were calculated for the bacterial file and chloroplast file and the figures and statistical analysis were processed on each site separately from this point. Note: given the consistency of the number of reads across replicates for each extraction volume (Figure S2), we anticipate that a sequencing run consisting of samples of the same microvolume will produce a more homogenous sequencing output.

**Metagenome Sequencing**

Metagenomes were sequenced at the Australian Centre for Ecogenomics (ACE) at the University of Queensland. Metagenomic libraries were prepared using the Nextera Flex library preparation kit (Illumina Inc., USA). DNA was amplified following the Illumina protocol, with slight modifications to the protocol for the low-input samples. Briefly, metagenomic library was produced using all available low input sample (7-9 µL), which was then diluted using PCR grade cross-linked water to 20 µl before undergoing a modified 20 cycle protocol was used based on previous testing and optimization (6). While the 2 L bulk samples were diluted 1:5 to 20 µL before undergoing only 12 PCR cycles. All samples were then sequenced on a NextSeq500 platform 2× with 150 bp High Output v.2 run chemistry.

Four sets of triplicate blanks (one set without any additional input volume, and another three with cross-linked MilliQ water added at volumes of 100 µL, 10 µL, and 1 µL), were all processed concomitantly to identify potential contamination.

Contaminant reference library

To remove extraction contaminant, reads from the blanks were assembled, and the resulting contigs were used to create a library of contaminants for each extraction method. Briefly, raw reads from the blanks were quality filtered, adapter removed using Trimmomatic (9) and assembled with metaSPAdes (10). The resulting assemblies were concatenated to create a contaminant library for each extraction type (physical lysis, chemical lysis or 2 L extraction). Furthermore, contigs were binned using Metabat2 in Anvio (v 6.2) to identify possible source of contaminations (11,12). For the chemical lysis in particular two almost complete MAGs were retrieved (completion > 50%) belonging to *Shewanella* and *Pseudomonas* (GTDBtk) (13). These two MAGs accounted for more than 80% of the reads found in the blanks.

Contaminants removal from samples

Adapter trimmed and quality filtered reads (9) for each sample were stringently quality filtered by removing any reads that mapped (bbmap, from the BBMap suite (14); <https://sourceforge.net/projects/bbmap/>) to assembled contigs for the corresponding extraction blanks (i.e., samples from the chemical lysis extraction were quality controlled by removing any reads that mapped to a contigs in the contaminant library from the chemical lysis extraction blanks). The amount of reads eliminated by this stringent quality control step ranged from less than 1% (for the physical lysis extraction and bulk 2 L) to up to 40% (for the 1 µL chemical lysis extraction) (Figure S8 and Table S13).

Number of unique reads per sample

Given that the number of amplification cycles were increased to 20 for the low input samples, the number of read duplicates (that can interfere with reads assemblies) was assessed for all samples. Dedupe, from the BBMap suite (14), was used to remove the duplicated reads (Figure S8).

Metagenome Assembly and functional annotation

Quality filtered reads for each sample were assembled using metaSPAdes (10), and assemblies were quality checked using metaQuast (15). Assemblies were evaluated for both the “blank-removed” reads and for the “blank-removed and deduplicated” reads (Table S13). Only the contigs assembled from the “blank-removed and deduplicated” reads were used for further analysis because of the better assembly performance compared to the reads with duplicates. Open reading frames were identified using Prodigal in metagenome mode (16). ORFs were then clustered at 95% identity using CD-HIT with the following parameters (-c 0.95 -T 16 -G 0 -aS 0.9 -g 1 -r 1 -d 0) and the function assigned with eggnog v.5 (17,18).

Functional profiles

Quality filtered reads for each sample were aligned against the gene catalogue using BBMap. To account for difference in sequencing depth, gene counts were then normalized per sample based on the total number of reads. The final functional profile table was produced by summing the normalized KO hits per sample.

Insert size

The files generated from the BBMap mapping were utilized to calculate the average insert size leveraging the BBMap suite program readlength.sh. Insert size was defined as the average length of merged reads mapping back to the assembly or genes catalogue.

Metagenome assembled genomes (MAGs)

The unique and adapter-free reads for the quadruplicate of each treatment were co-assembled using MegaHIT (19). The resulting contigs were binned using Metabat2 in Anvio (v 6.2) (11,12) as above and the MAGs refined in Anvio and quality checked with CheckM . Taxonomy of each MAG was assigned with GTDB-Tk (13). We used fastANI to assess the degree of similarity between the assembled MAGs of good quality (completion >50%, redundancy <20%) for each extraction and volume (Table S17). MAGs with a fastANI score above 0.99 were considered the same (Table S18) (20).

**Data Analysis**

Multidimensional Scaling

To explore the differences between the microbial assemblage composition and abundances identified by amplicon sequencing, we square root-transformed the rarefied amplicon abundance table and used the *metaMDS* function in *R* to compute Bray-Curtis distances of all comparisons and preformed a non-metric multidimensional scaling (nMDS) to visualize how both extraction type and sample input volume influenced the overall microbial composition of the samples. The same procedure was applied to metagenome functional profiles. Briefly, we square root-transformed the normalized KO abundance table and used the *metaMDS* with the same parameters, as per the amplicon nMDS analysis. We also computed the ordinations without square root transformation for each site (Figure S4: amplicon; and Figure S11 and S12: metagenomes).

Statistical Analysis

To determine if statistical differences existed at the taxonomic and functional levels between extraction types and volumes, permutational multivariate analysis of variance (PERMANOVA) were computed in *R* using the *pairwise.adonis* function. The following parameters were used throughout (p.adjust.m = "*bonferroni*").

To determine the variability between replicates depending on extraction types or volumes, we first calculated their dispersion using the *R* functions *betadisper* followed by *permutest*. Dispersion values were then compared statistically using an analysis of variance (ANOVA; using the *aov* function), followed by multiple comparisons with Bonferroni correction (using the *PostHocTest* function). The dispersion of the Shannon diversity indexes derived from the amplicon analysis was also compared between extraction types and volumes (ANOVA; using the *aov* function), followed by multiple comparisons with Bonferroni correction (using the *PostHocTest* function).

Correlations

To determine the degree of similarity between the various extraction types and volumes, we used the *rcorr* function in *R* to calculate the Spearman’s correlation (i) of all ASVs after rarefying for the amplicon dataset (all correlations were statistically significant p <0.0001) (Figure S5); and (ii) of all the annotated functional genes from the metagenomes (Figure 2b). The Spearman’s correlation for the metagenomic data was calculated using the same function, but on a subset of the normalized functional genes (number of raw reads per gene across the whole dataset > 10).

Suggested Modifications

Based on our results, we anticipate that specific modifications to the chemical lysis procedure will further decrease the level of contamination derived from the reagents, which will likely facilitate genomes assembly and the extraction of nanolitre volumes. The suggested modifications include: (i) cross-linking the Lysozyme, Proteinase-K and SDS for up to 1 hr on ice prior to use; (ii) the replacement of cross-linked MilliQ by DNAse/RNAse-free water (UltraPure Distilled Water, Invitrogen, USA); (iii) the use of the full eluted volume from 1 µL extraction (10 µL) for library preparation (Nextera Flex), thereby doubling the DNA input compared to what was used in the current work (where we only used 5 µL of the eluted DNA).

Data Availability

Raw reads are available from NCBI under project PRJNA766133, the accession numbers for the amplicon data are SAMN21619120-SAMN21619181, and those for the metagenomic data are SAMN22233957-SAMN22234011.

**References**

1. Woyke T, Sczyrba A, Lee J, Rinke C, Tighe D, Clingenpeel S, et al. Decontamination of MDA reagents for single cell whole genome amplification. *PLoS One*. 2011;6(10):2–6.

2. Yang B, Wang Y, Qian PY. Sensitivity and correlation of hypervariable regions in 16S rRNA genes in phylogenetic analysis. *BMC Bioinformatics*. 2016;17(1).

3. R Core Team. R: A language and environment for statistical computing. 2020.

4. Callahan BJ, McMurdie PJ, Rosen MJ, Han AW, Johnson AJA, Holmes SP. DADA2: High-resolution sample inference from Illumina amplicon data. *Nat Methods*. 2016;13(7):581–3.

5. Martin M. Cutadapt removes adapter sequences from high-throughput sequencing reads. *EMBnet J*. 2011;(17):10–2.

6. Rinke C, Low S, Woodcroft BJ, Raina JB, Skarshewski A, Le XH, et al. Validation of picogram- and femtogram-input DNA libraries for microscale metagenomics. *PeerJ*. 2016;(9):1–28.

7. McKnight DT, Huerlimann R, Bower DS, Schwarzkopf L, Alford RA, Zenger KR. Methods for normalizing microbiome data: An ecological perspective. *Methods Ecol Evol*. 2019;10(3):389–400.

8. Weiss S, Xu ZZ, Peddada S, Amir A, Bittinger K, Gonzalez A, et al. Normalization and microbial differential abundance strategies depend upon data characteristics. *Microbiome*. 2017;5(1):1–18.

9. Bolger AM, Lohse M, Usadel B. Trimmomatic: A flexible trimmer for Illumina sequence data. *Bioinformatics*. 2014;30(15):2114–20.

10. Eliseev A, Gibson KM, Avdeyev P, Novik D, Bendall ML, Pérez-Losada M, et al. Evaluation of haplotype callers for next-generation sequencing of viruses. *bioRxiv*. 2019;1–32.

11. Kang DD, Li F, Kirton E, Thomas A, Egan R, An H, et al. MetaBAT 2: An adaptive binning algorithm for robust and efficient genome reconstruction from metagenome assemblies. *PeerJ*. 2019;2019(7):1–13.

12. Eren AM, Kiefl E, Shaiber A, Veseli I, Miller SE, Schechter MS, et al. Community-led, integrated, reproducible multi-omics with anvi’o. *Nat Microbiol*. 2021;6(1):3–6.

13. Chaumeil PA, Mussig AJ, Hugenholtz P, Parks DH. GTDB-Tk: A toolkit to classify genomes with the genome taxonomy database. *Bioinformatics*. 2020;36(6):1925–7.

14. Bushnell B. BBMap: A Fast, Accurate, Splice-Aware Aligner. 2014.

15. Mikheenko A, Saveliev V, Gurevich A. MetaQUAST: Evaluation of metagenome assemblies. *Bioinformatics*. 2016;32(7):1088–90.

16. Doug Hyatt, Gwo-Liang Chen, Philip F LoCascio, Miriam L Land, , Frank W Larimer LJH. Prodigal: prokaryotic gene recognition and translation initiation site identification. *Nat Commun*. 2010;6(1):1–8.

17. Li W, Godzik A. Cd-hit: A fast program for clustering and comparing large sets of protein or nucleotide sequences. *Bioinformatics*. 2006;22(13):1658–9.

18. Huerta-Cepas J, Szklarczyk D, Heller D, Hernández-Plaza A, Forslund SK, Cook H, et al. EggNOG 5.0: A hierarchical, functionally and phylogenetically annotated orthology resource based on 5090 organisms and 2502 viruses. *Nucleic Acids Res*. 2019;47(D1):D309–14.

19. Li D, Liu CM, Luo R, Sadakane K, Lam TW. MEGAHIT: An ultra-fast single-node solution for large and complex metagenomics assembly via succinct de Bruijn graph. *Bioinformatics*. 2015;31(10):1674–6.

20. Jain C, Rodriguez-R LM, Phillippy AM, Konstantinidis KT, Aluru S. High throughput ANI analysis of 90K prokaryotic genomes reveals clear species boundaries. *Nat Commun*. 2018;9(1):1–8.

**Supplementary Figures**

**Figure S1:** **Microvolume DNA extraction methods for extracting trace DNA samples**. Microvolume samples can be extracted using two new methods: (a) Physical Lysis, relying on an alkaline buffer (pH =12) incubated with the cells for 10 minutes, followed by a freeze-thaw step to lyse the cells. (b) Chemical Lysis, relying on lysozyme and sodium dodecyl sulfate (SDS) to lyse the cells. (c) These two different lysis protocols are followed by a DNA clean-up step using AMPure beads (Beckman Coulter, USA), two successive EtOH washes, followed by elution of the pure DNA.


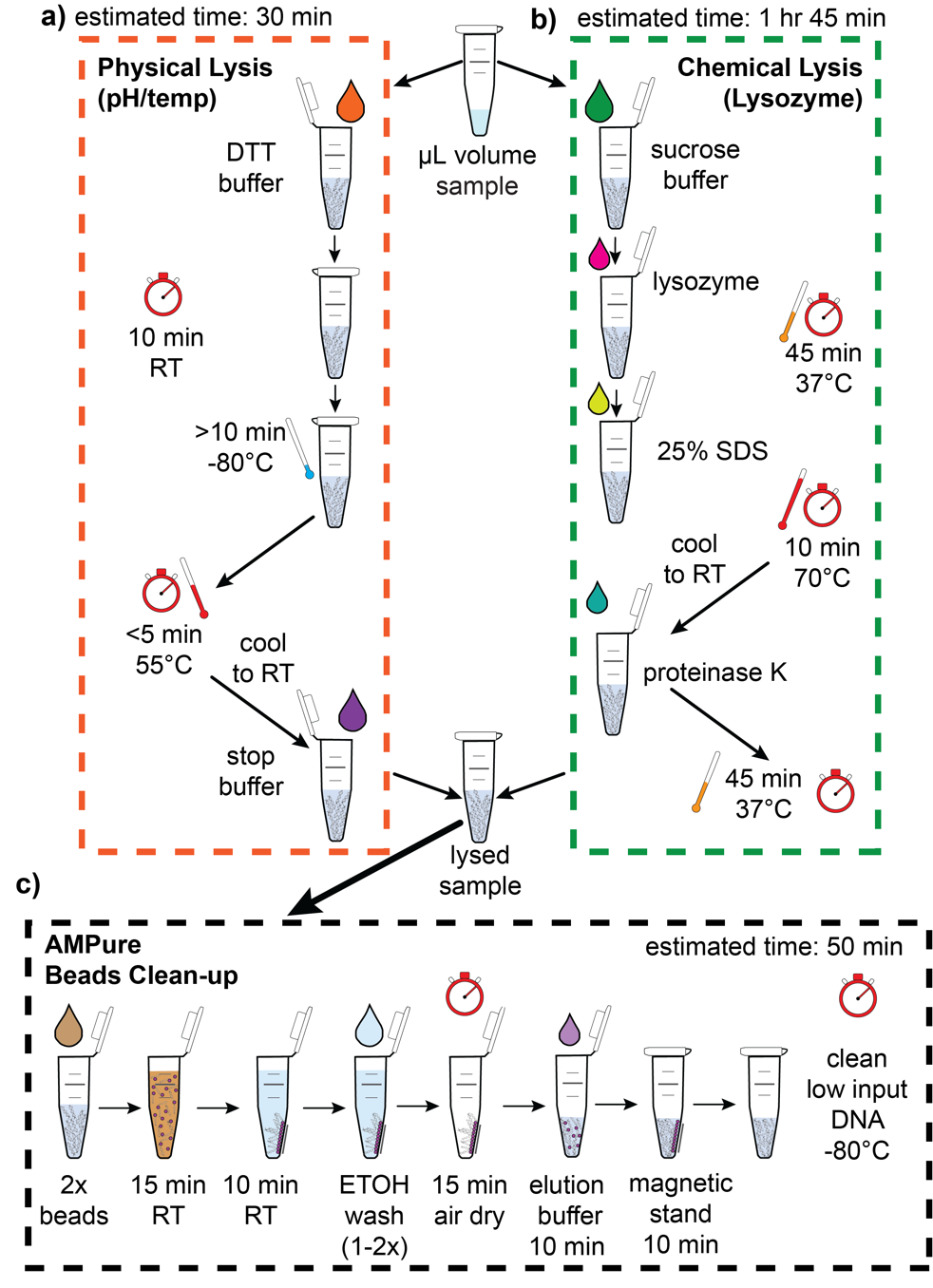


**Figure S2:** **Number of** **amplicon reads per sample following processing through the DADA2 pipeline**. Number of reads for the (a) oceanic and (b) coastal site. Samples were extracted using either: (i) a PowerWater kit (PW) (2 L of seawater filtered on 0.22 µm), (ii) the Physical Lysis (100 µL, 10 µL, and 1 µL), or the Chemical Lysis extraction (100 µL, 10 µL, and 1 µL).


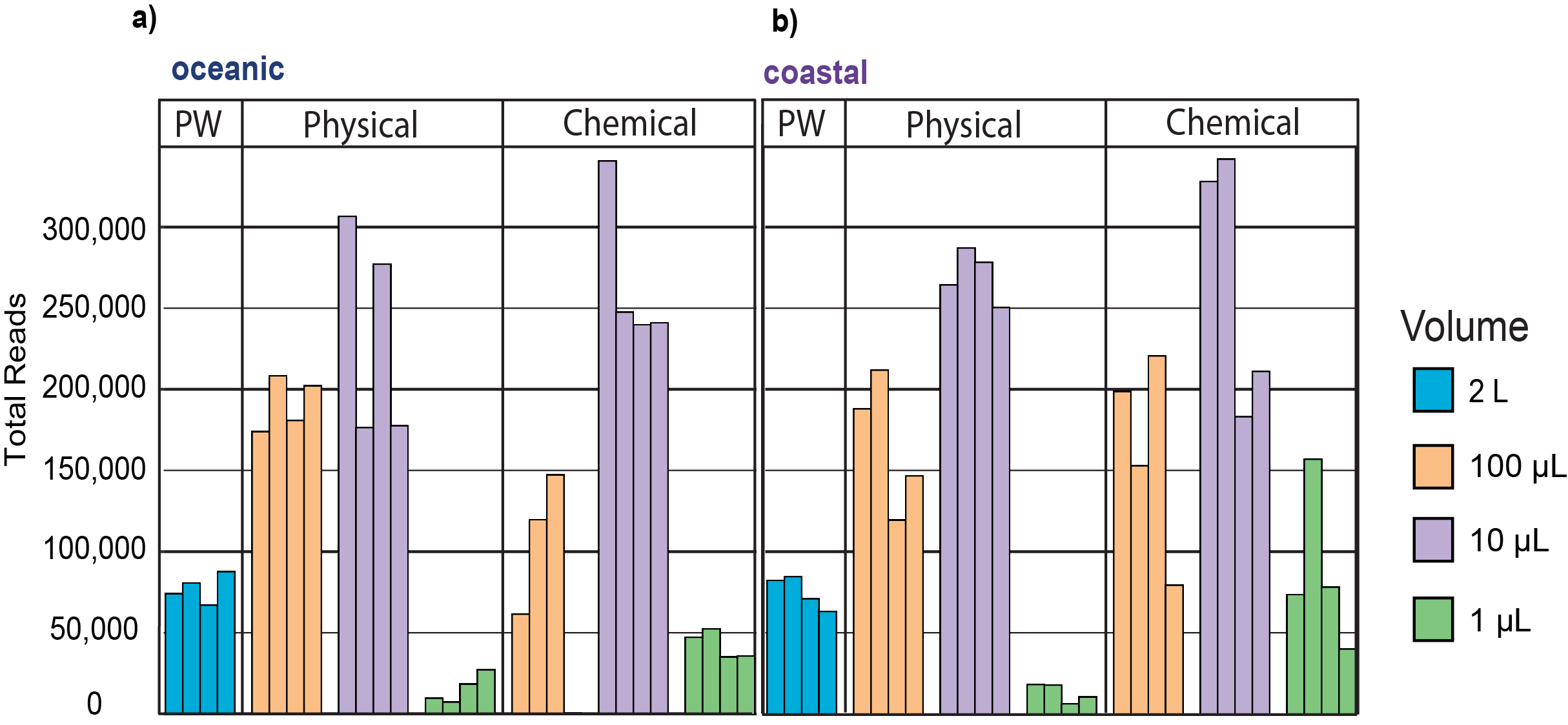


**Figure S3:** **Non-metric multidimensional scaling of the amplicon sequencing derived from the oceanic and coastal sites after rarefying at a range of depths**. (a) 5,500 reads, (b) 10,000 reads, (c) 20,000 reads, (d) 30,000 reads. This range resulted in the full inclusion of all 1 µL physical lysis extraction replicates when rarefied at 5,500 reads (which was equivalent to 96% of the lowest read depth), to the complete exclusion of these replicates when rarefied at 30,000 reads.


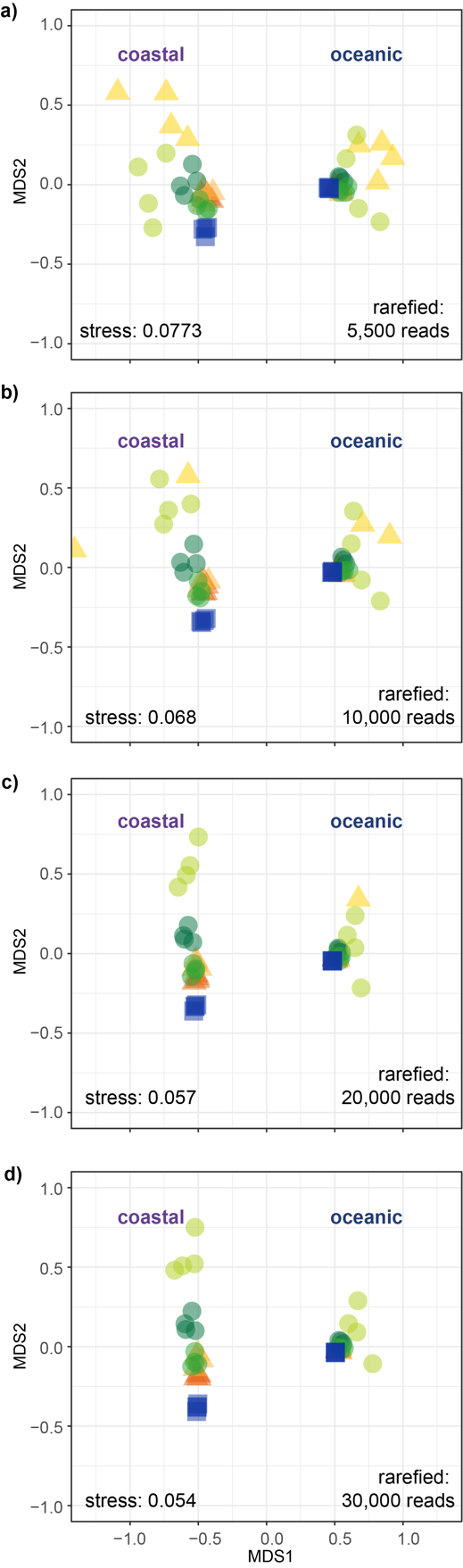


**Figure S4: Non-metric multidimensional scaling of relative abundance of all extraction types and volumes for each site**. Non-metric multidimensional scaling (nMDS) plots based on Bray-Curtis distance between square root-transformed samples for the (a) oceanic and (b) coastal sites. nMDS plots without square root transformation for the (c) oceanic and (d) coastal sites (matches Figure 1b, but sites are split into separate ordinations and shown both with and without square root transformation).


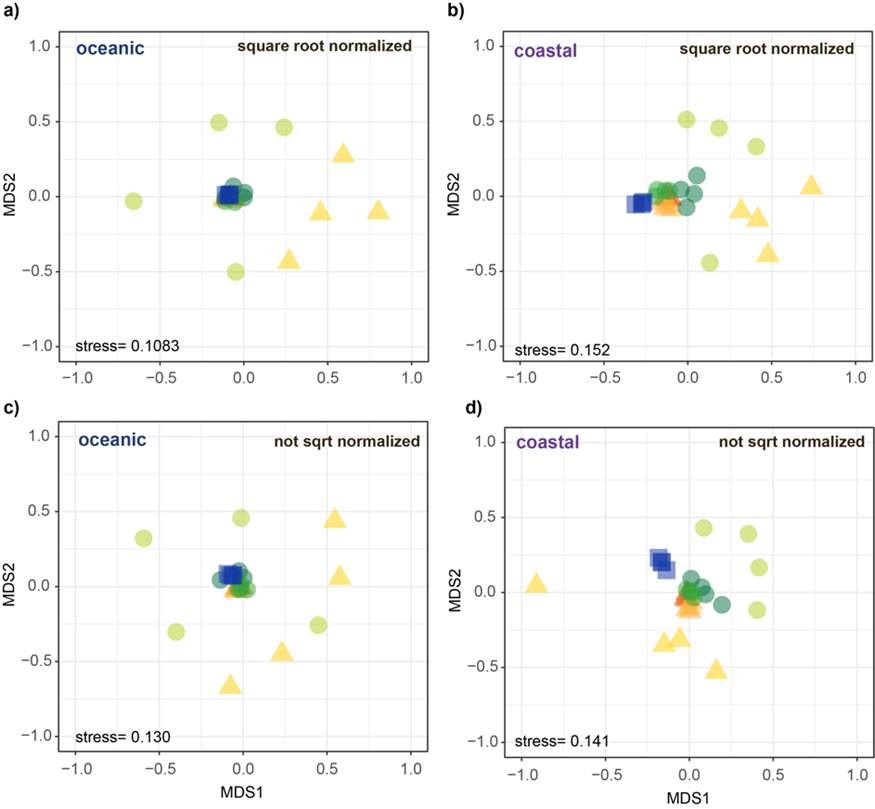


**Figure S5:** **Spearman’s correlation of the amplicon consortia in the different extraction types and volumes for the (a) oceanic and (b) coastal sites.** All correlations were based on average reads of bacterial 16S rRNA gene after rarefaction to 5,500 reads. All correlations shown were statistically significant (*p*<0.0001), which reveals the high similarity in community composition regardless of extraction type or extraction volume.


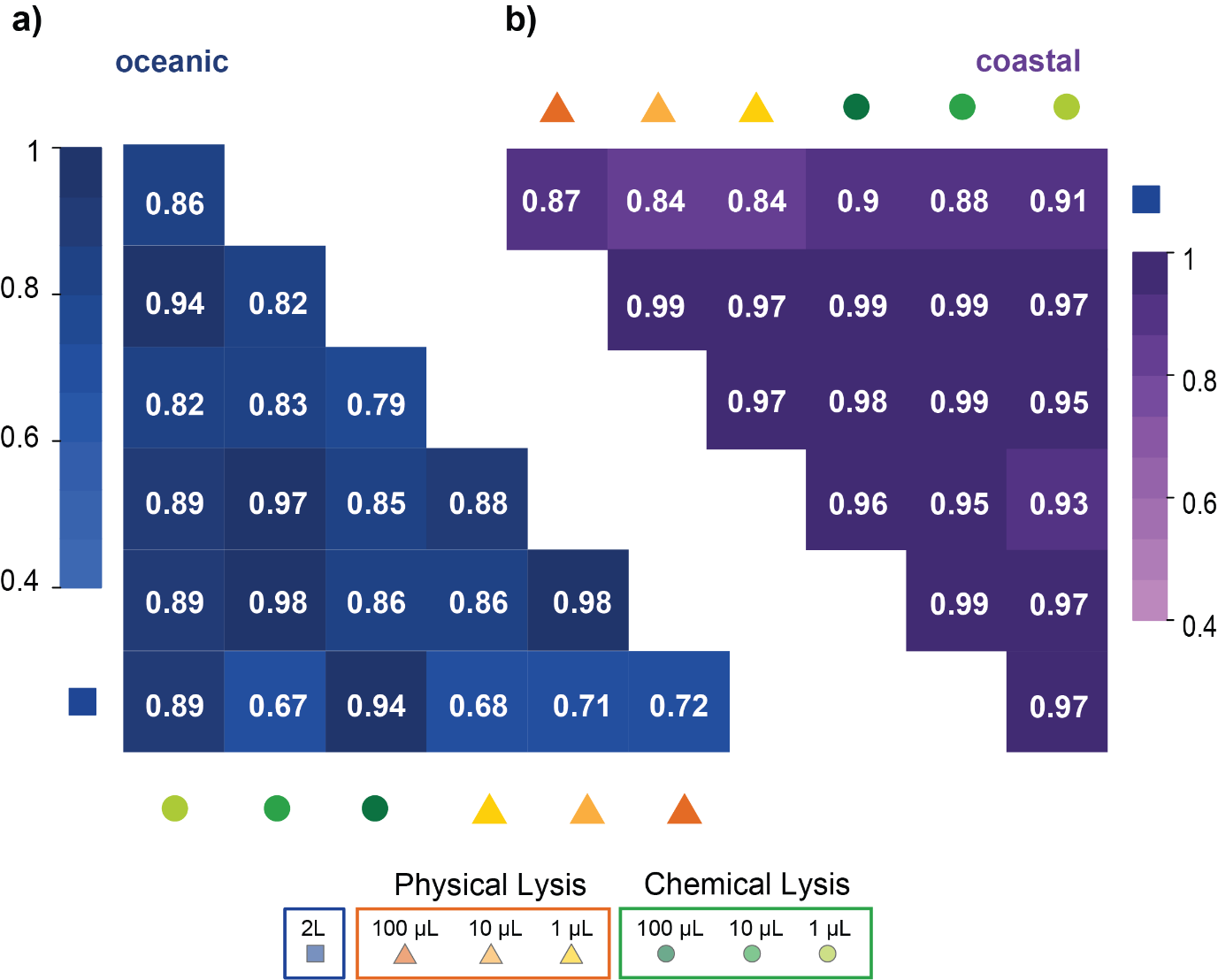


**Figure S6: Oceanic site average 16S rRNA amplicon correlation plots of each extraction type and volume against the PowerWater 2 L extraction** (on the x-axis). Pearson correlations of the relative abundance of ASVs at the oceanic site between the 2 L extraction and (a) physical lysis 100 µL; (b) chemical lysis 100 µL; (c) physical lysis 10 µL; (d) chemical lysis 10 µL; (e) physical lysis 1 µL; (f) chemical lysis 1 µL. Linear regression in blue, Pearson correlation *R* values and number of ASVs common to both sample volumes are shown on each respective plot. Dots are sized based on the average relative abundance of the ASV in the 2 L sample. Average number of ASVs present in each sample noted for each volume, and the number of ASVs shared between the microvolume extractions and the 2L extraction (grey dots) is noted for each comparison (as n=#). Green dots on the y-axis represent ASVs only found in the respective micro-volume extraction and red dots on the x-axis represent ASVs only found in the 2L extraction.


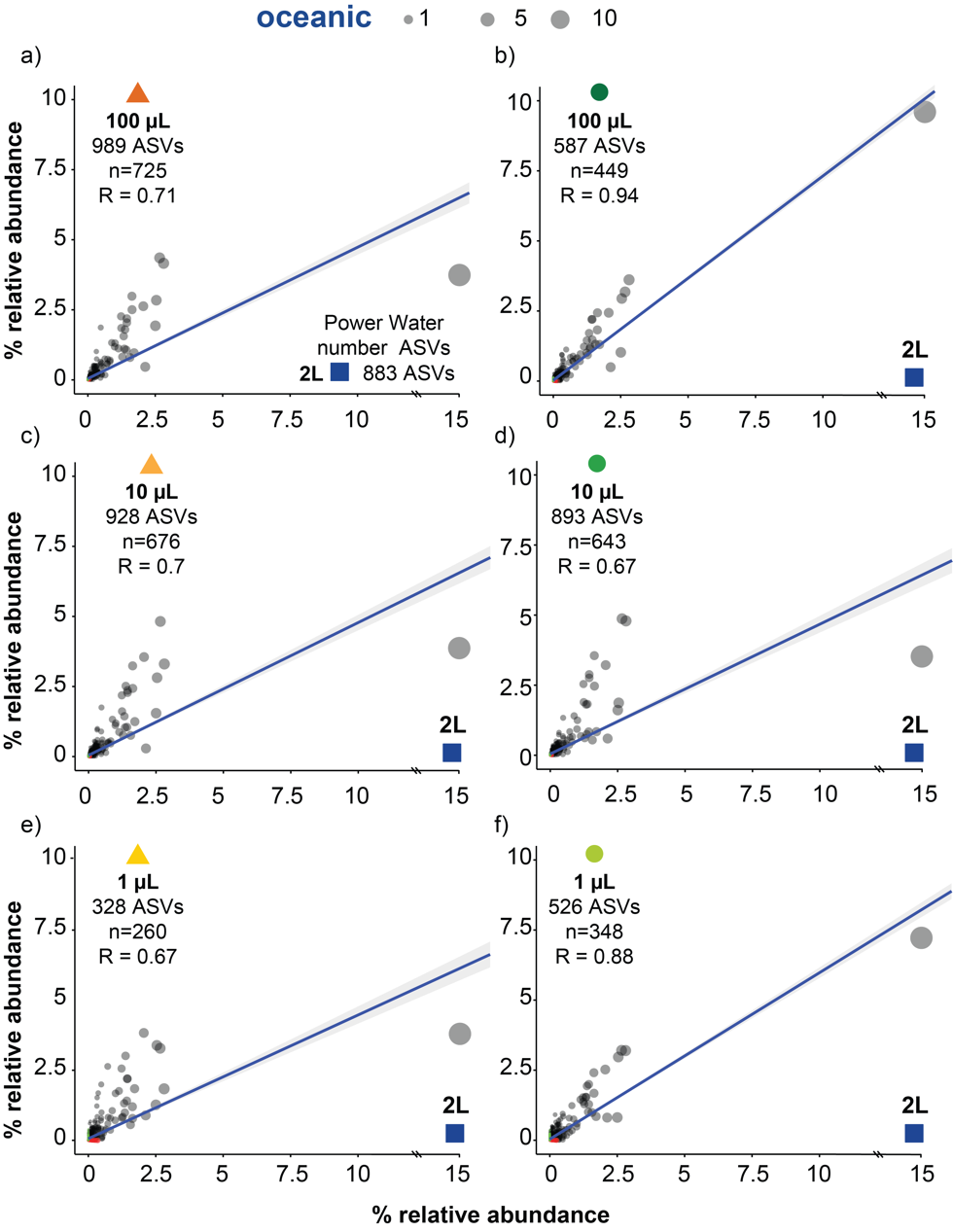


**Figure S7: Coastal site average 16S rRNA amplicon correlation plots of each extraction type and volume against the PowerWater 2 L extraction** (on the x-axis). Pearson correlations of the relative abundance of ASVs at the oceanic site between the 2 L extraction and (a) physical lysis 100 µL; (b) chemical lysis 100 µL; (c) physical lysis 10 µL; (d) chemical lysis 10 µL; (e) physical lysis 1 µL; (f) chemical lysis 1 µL. Linear regression in blue, Pearson correlation *R* values and number of ASVs common to both sample volumes are shown on each respective plot. Dots are sized based on the average relative abundance of the ASV in the 2 L sample. Average number of ASVs present in each sample noted for each volume, and the number of ASVs shared between the microvolume extractions and the 2L extraction (grey dots) is noted for each comparison (as n=#). Green dots on the y-axis represent ASVs only found in the respective micro-volume extraction and red dots on the x-axis represent ASVs only found in the 2L extraction.


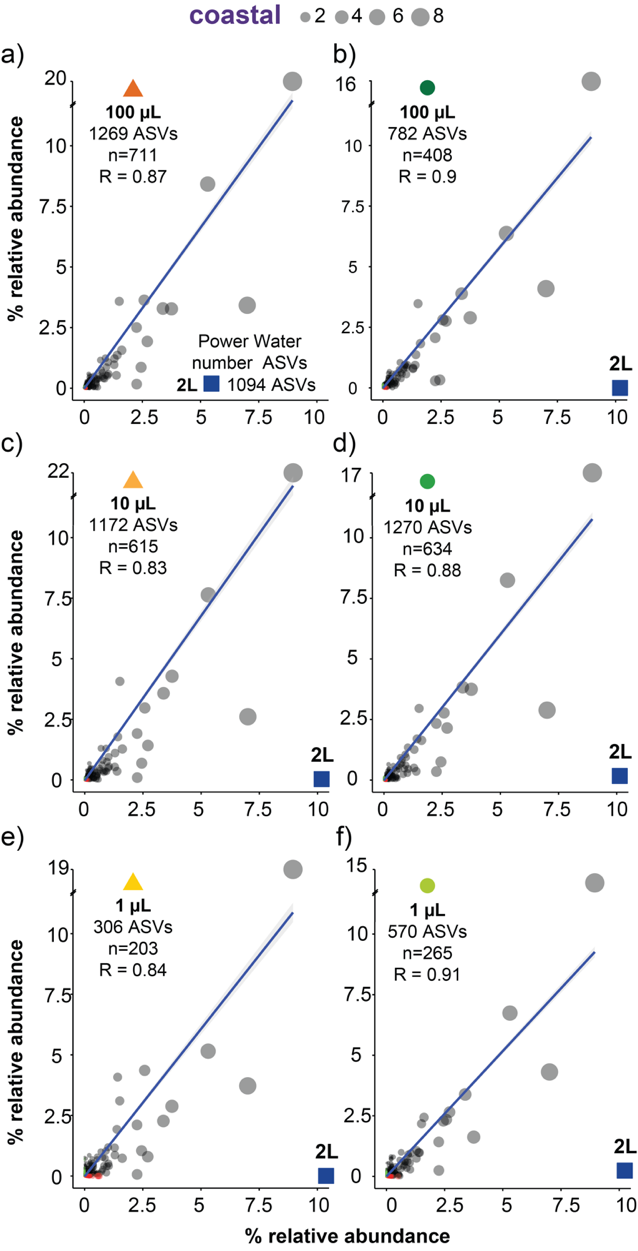


**Figure S8: Sequencing yield and library quality of the metagenomes**. The bar plot indicates the number of (a) raw reads, (b) unique reads (calculated with the calculateunique.sh script of the BBMap package) after the removal of reads that mapped to the specific extraction contaminant library, and (c) number of contigs (over 500 bp long), which were identified in each extraction type and volume: PowerWater (PW), physical and chemical lysis.


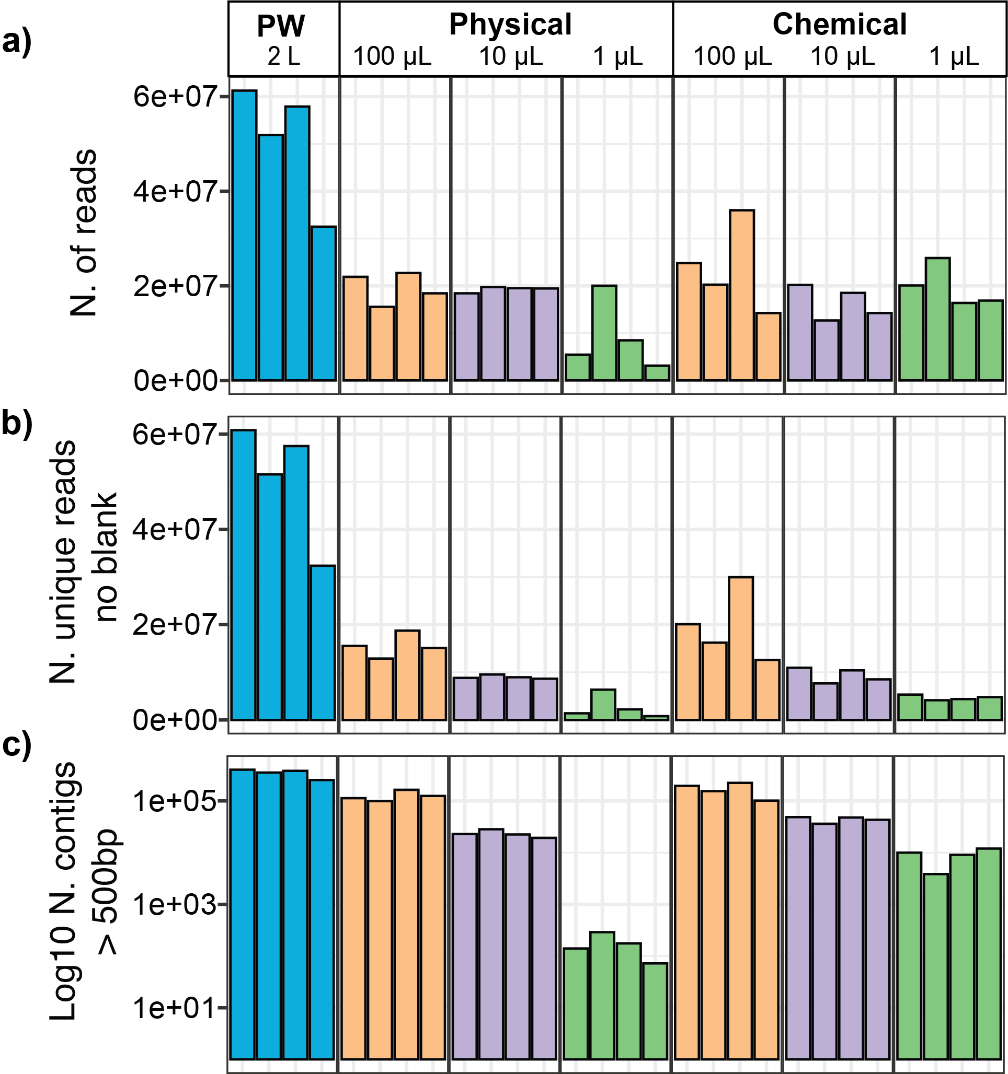


**Figure S9: Average reads insert size (bp) for each of the different extraction** **compared to the percentage of unique reads after blank removal per extraction volume**. Insert size is relative to the average length of trimmed merged reads that mapped back to the assembly, extracted from the sam file obtained with BBmap.


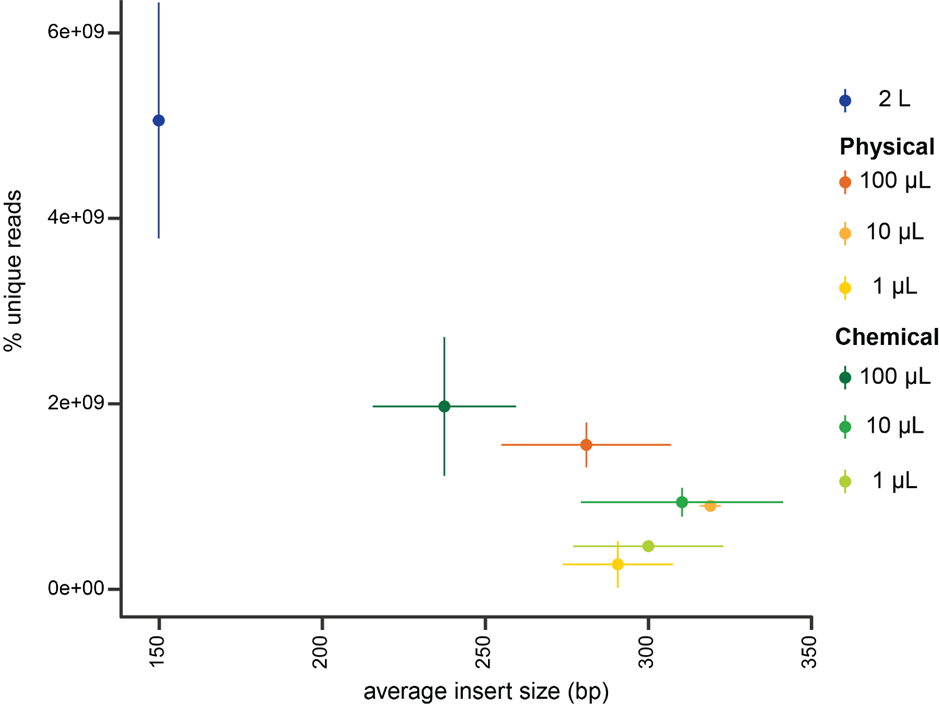


**Figure S10: Dispersion between replicates of the functional composition of the seawater metagenomes from the oceanic site** **using the different extraction methods and seawater volumes**. Dispersion of the replicates corresponding to Figure 2a. Dispersions that were statistically different (*p*<0.05) from the 2 L are marked (*), all statistics for dispersions (Table S28).


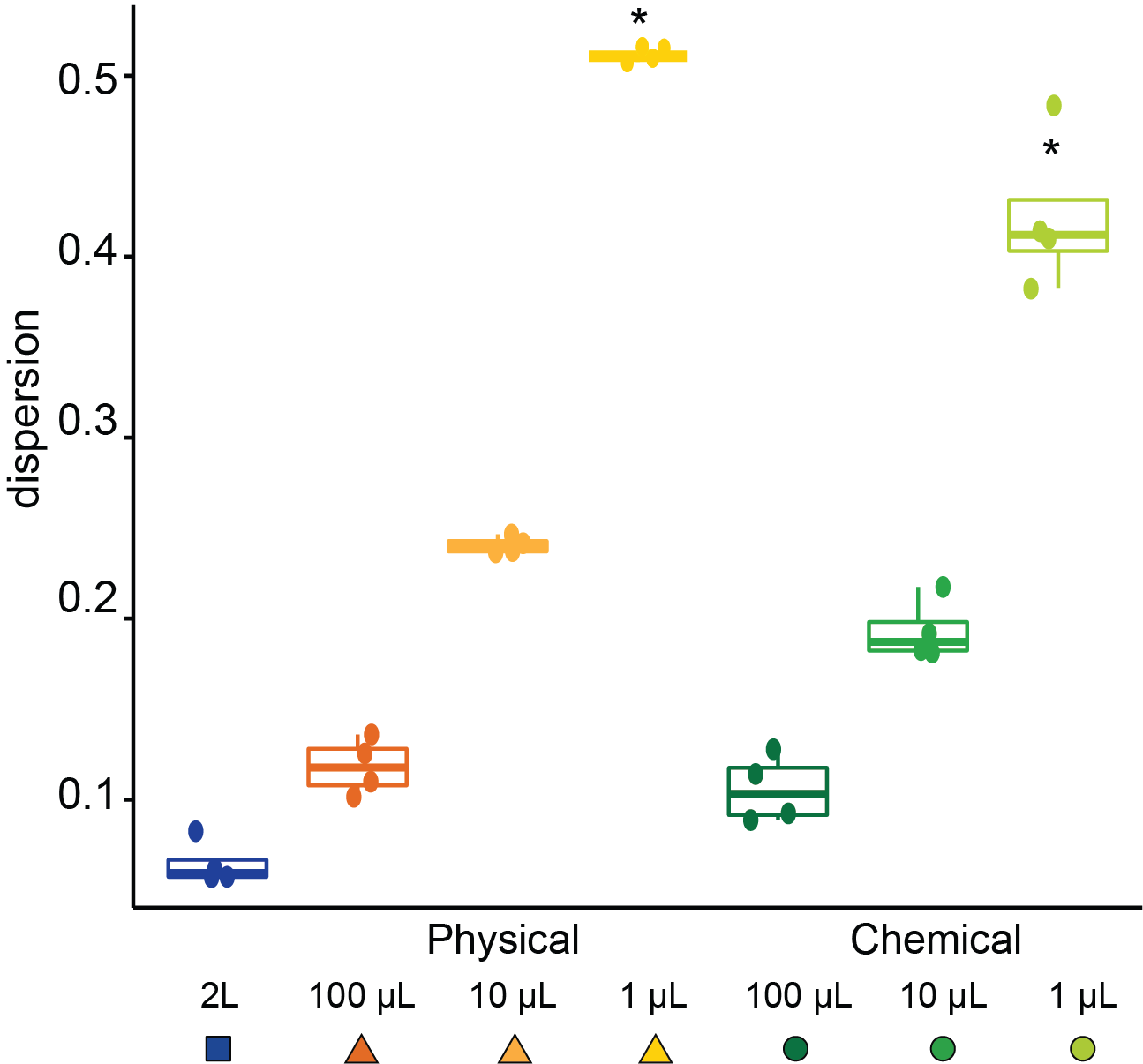


**Figure S11: Non-metric multidimensional scaling of functional composition of the seawater metagenomes from the oceanic site** **(using all genes >100 reads)**. Non-metric multidimensional scaling (nMDS) plot based on Bray-Curtis distance between samples of the oceanic site without square root transformation (matches Figure 2a, but without square root transformation).


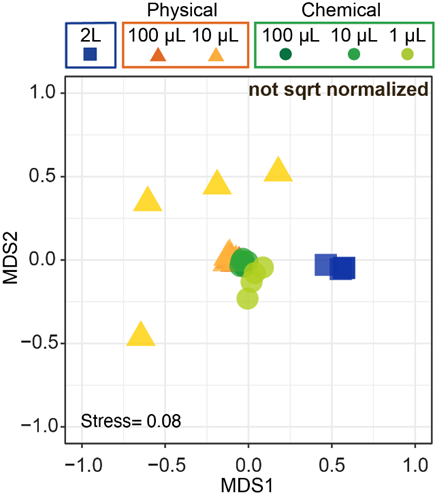


**Figure S12: Functional composition of the seawater metagenomes from the oceanic site** **comparing the different extraction methods and seawater volumes (using all genes >100 reads)**. (a) Square root transformed non-metric multidimensional scaling (nMDS) plot based on Bray-Curtis distance between all genes (with at least 100 reads; PERMANOVA results found in Table S15). (b) nMDS plot based on Bray-Curtis distance between all genes (with no square root transformation; with at least 100 reads). (c) Metagenomic genes matching Figure 2c, but showing all genes present without a threshold. The full legend for panel (c) can be found in Table S16.


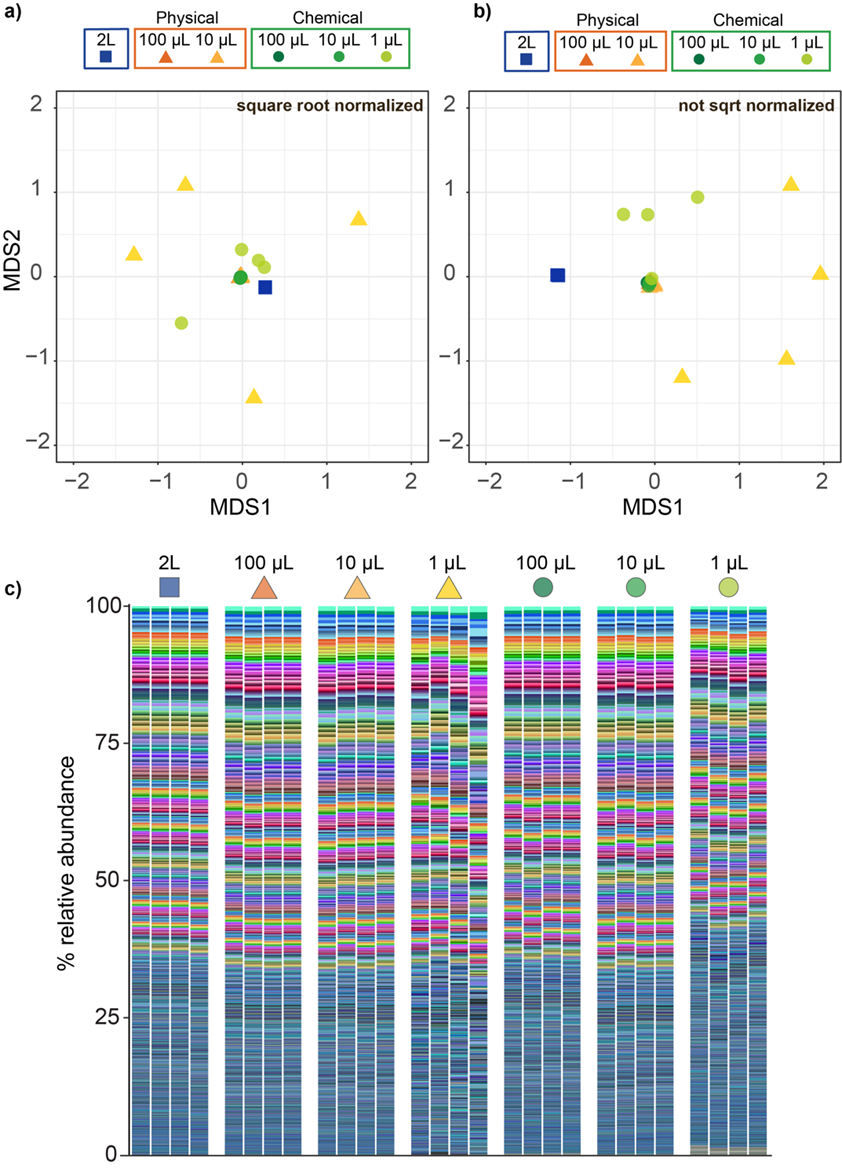


**Figure S13: Correlations of the func­­tional genes for each extraction method and volume against the PowerWater 2 L extraction** (on the x-axis). Reads mapping for each functional gene was normalized based on the total number reads per samples. The normalized gene count was then averaged across replicates. Linear regression in blue, Pearson correlation *R* values and number of functional genes common to both sample volumes are shown on each respective plot. Green dots on the y-axis are functional genes only found in the respective microvolume extraction and red dots on the x-axis are functional genes only found in the 2L extraction (missing from the corresponding microvolume extraction).


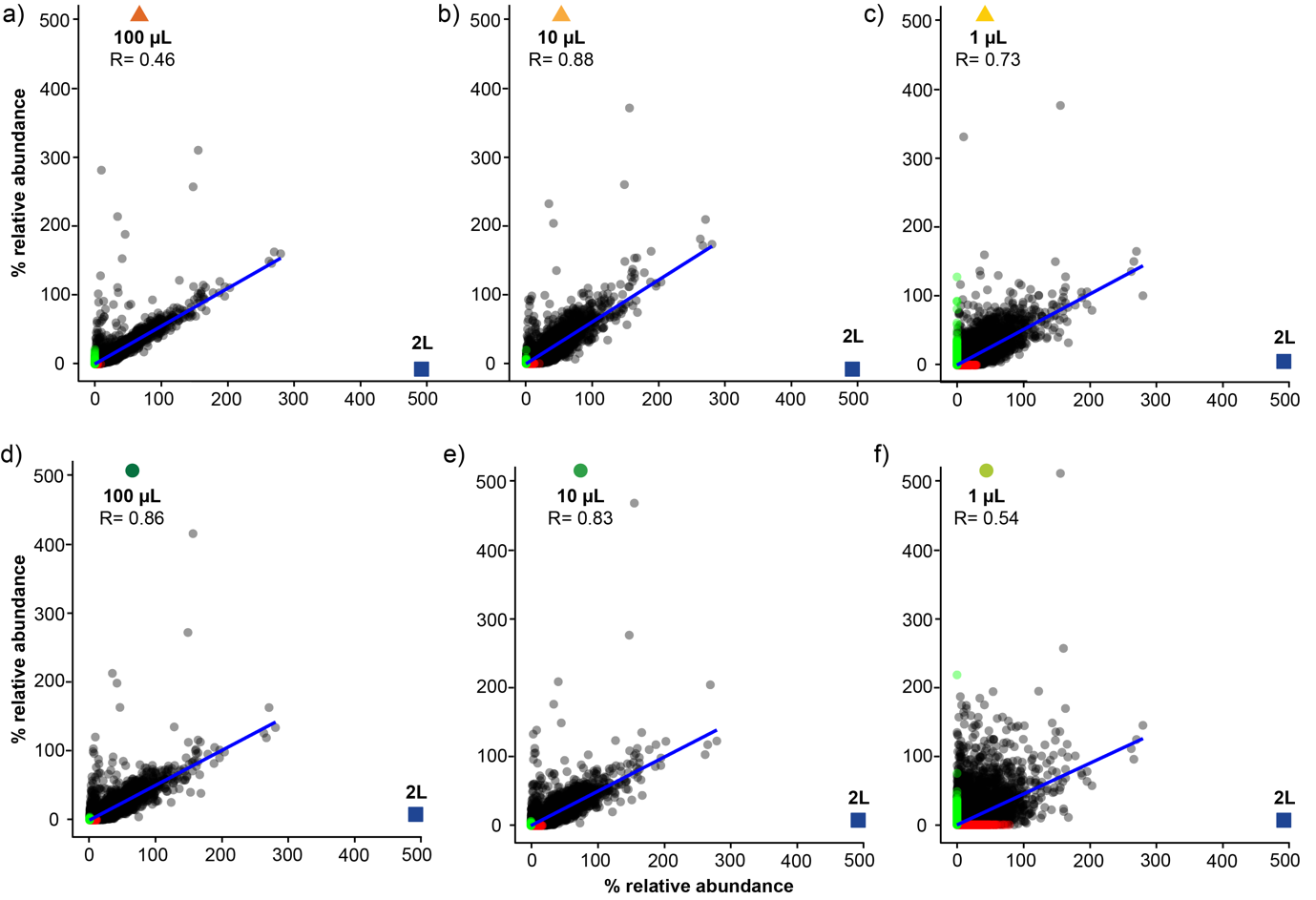


**Figure S14:** **Dispersion of the replicates increased as volume sampled decreased**. The variability of the replicates increased significantly (*p*<0.05) at lower volumes compared to the 2L extraction for both the oceanic (a) and coastal sites (b). Microvolume extractions that display a significantly greater dispersion of their replicates compared to the 2 L extraction are noted on the graph (**). All statistical results are present in Supplementary Tables S19 and S20.


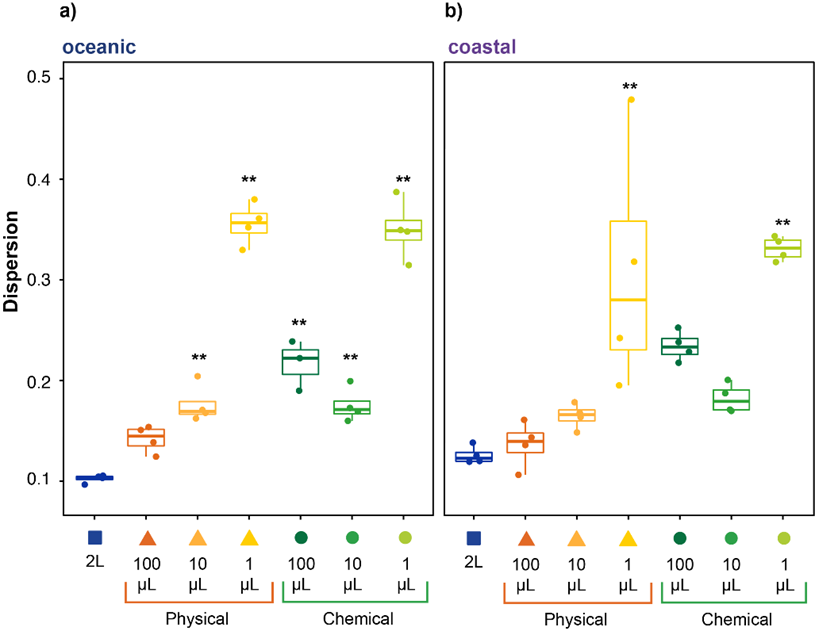


**Figure S15:** **Variability and dispersion of chloroplast diversity increases as extracted volume decreases**. Taxonomic composition of the chloroplast sequences at the (a) oceanic and (b) coastal site. (c) Chloroplast sequences within each site were statistically indistinguishable (PERMANOVA, *p*>0.05, Tables S23-S24), however both sampling sites were statistically different (PERMANOVA, p<0.001, Table S25). (d) Dispersion of the replicates. Dispersions that were statistically different from the 2 L extraction (*p* <0.05) are noted with an asterisk (*). All statistical results are present in Tables S26 and S27.


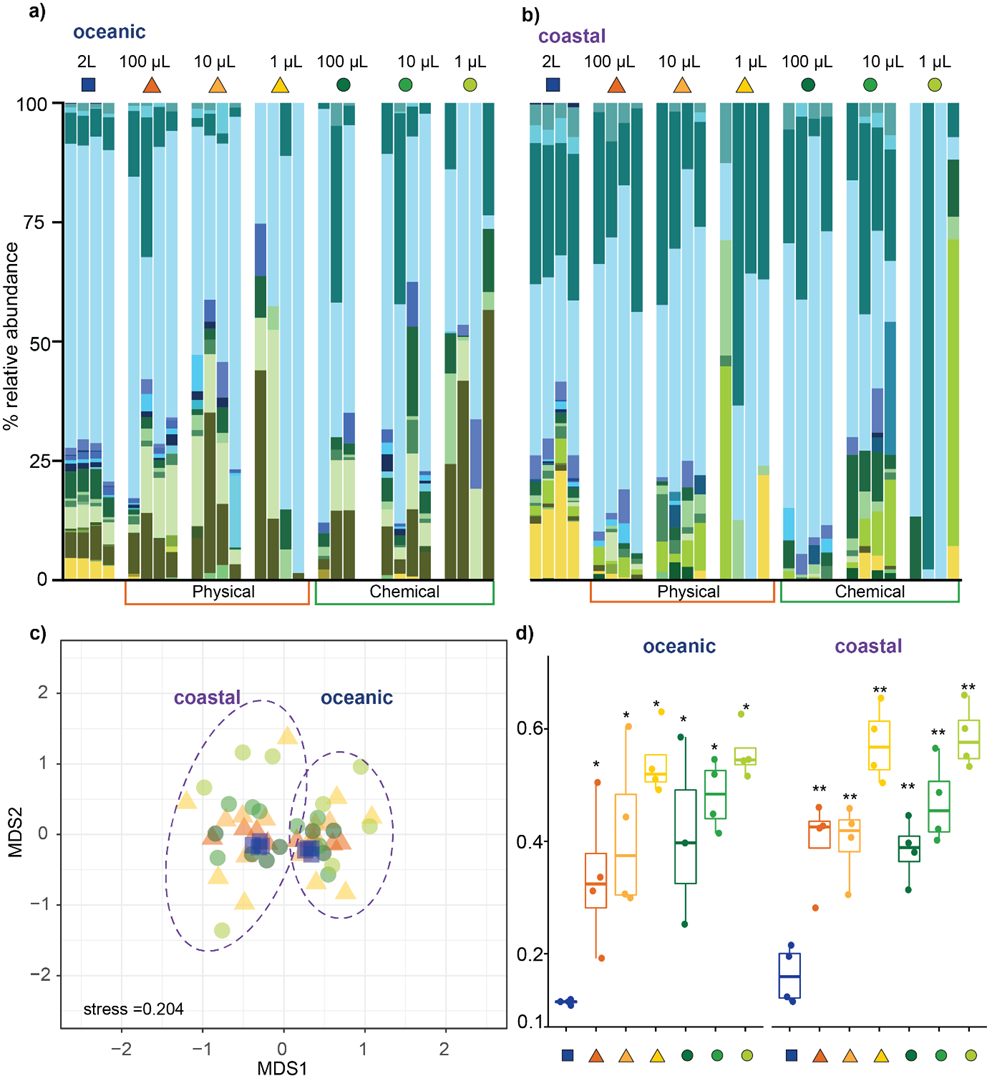


**Figure S16:** **Shannon diversity indexes of the different extraction types and volumes**. Shannon diversity index of bacterial 16S rRNA amplicon sequences at the (a) oceanic and (b) coastal sites. Alpha diversity measurements that were statistically different from the 2 L extraction (*p* <0.01) are noted with (*), (*p* <0.001) are noted with (**). All statistical results are present in Tables S21 and S22.


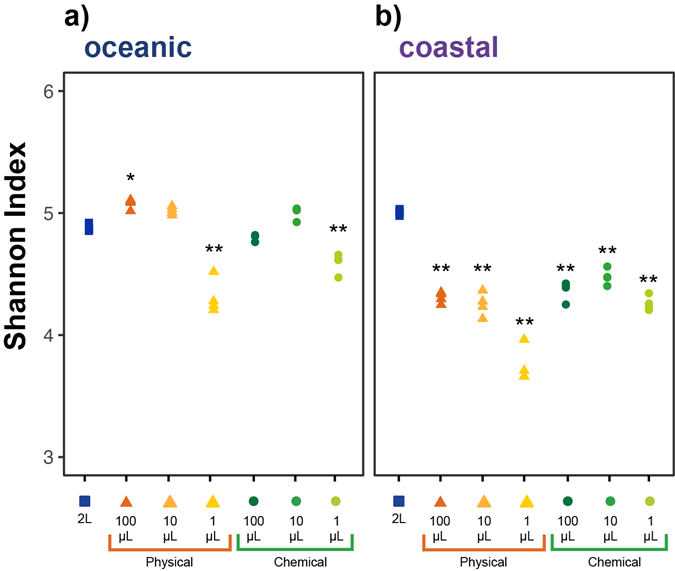


**Supplementary Tables**

**Table S1:** Flow cytometry counts of the number of cells present in the bulk seawater at the oceanic and coastal sites at the time of extraction (n=3). This table also presents the expected number of cells at the different volumes extracted: 2 L, 100 µL, 10 µL, 1 µL.

**Table S2:** Volume required for the different components of the Physical Lysis extraction depending on the initial sample volume to be extracted (100 µL, 10 µL, 1 µL).

**Table S3:** Volume required for the different components of the Chemical Lysis extraction depending on the initial sample volume to be extracted (100 µL, 10 µL, 1 µL).

**Table S4:** Bacterial 16S rRNA V3-V4 amplicon primers (in bold) with adaptors.

**Table S5:** PCR master mix used for the initial 25 cycles to amplify the V3-V4 region of bacterial 16S rRNA gene. The reaction volume was 25 µL.

**Table S6:** Volume used for the different steps of the sequencing procedure (based on the initial volume extracted).

**Table S7:** Percentage of amplicon reads removed during the decontamination protocol.

**Table S8:** PERMANOVA comparing the taxonomic composition of the two sampling sites (using Bonferroni corrected *p* values). This table reports the statistical results from the data presented on Figure 1b. Data were rarefied to 5,500 reads and square root transformed.

**Table S9:** PERMANOVA comparing the taxonomic composition of each treatment at the oceanic site (using Bonferroni corrected *p* values). This table reports the statistical results from the data presented on Figure 1c. Data were rarefied to 5,500 and square root transformed.

**Table S10:** PERMANOVA comparing the taxonomic composition of each treatment at the coastal site (using Bonferroni corrected *p* values). This table reports the statistical results from the data presented on Figure 1d. Data were rarefied to 5,500 and square root transformed.

**Table S11:** Colour palette used for Figure 1c. Amplicon sequence variants (ASVs) ordered from the most abundant (at the top). Only ASVs > 0.1% in at least one replicate are displayed in the bar plot. ASVs that were rare in the 2 L extractions (<0.1%) are coloured various shades of blue when present (>0.1% of any of the microvolume extractions). All ASVs absent from the 2 L extractions are coloured grey and are at the bottom of the plot if present (>0.1% of any of the microvolume extractions).

**Table S12:** Colour palette used for Figure 1d. Amplicon sequence variants (ASVs) ordered from the most abundant (at the top). Only ASVs > 0.1% in at least one replicate are displayed in the bar plot. ASVs that were rare in the 2 L extractions (<0.1%) are coloured various shades of blue when present (>0.1% of any of the microvolume extractions). All ASVs absent from the 2 L extractions are coloured grey and are at the bottom of the plot if present (>0.1% of any of the microvolume extractions).

**Table S13:** Metagenome statistics for each extraction type and volume using two different assembly methods (with and without duplicates removal). The latter assembly method performed better and was used for subsequent analyses.

**Table S14:** PERMANOVA comparing the functional potential of each treatment (using KEGG Orthology, KO) at the oceanic site (using Bonferroni corrected *p* values). This table reports the statistical results from the data presented on Figure 2a. Data were square root transformed.

**Table S15:** PERMANOVA comparing the functional genes distribution for each treatment at the oceanic site (using Bonferroni corrected *p* values). This table reports the statistical results from the data presented on Figure S12a. Data were square root transformed.

**Table S16:** Colour palette used for Figure 2c. KEGG Orthology (KOs) ordered from the most abundant (at the top). Only KOs > 0.1% in at least one replicate are displayed in the bar plot. KOs that were rare in the 2 L extractions (<0.1%) are coloured various shades of blue when present (>0.1% of any of the microvolume extractions). All KOs absent from the 2 L extractions are coloured grey and are at the bottom of the plot if present (>0.1% of any of the microvolume extractions).

**Table S17:** Metagenome assembled genomes (MAGs) co-assembled from the different extraction types and volumes. The MAGs highlighted in green meet the following criteria: completion >50% and redundancy <20%.

**Table S18:** MAGs similarity calculated with fastANI. MAGs with a similarity score >99% are considered as identical.

**Table S19:** ANOVA comparing the dispersion of the replicates for each extraction type and volume at the oceanic site (using Bonferroni corrected *p* values). This table reports the statistical results from the data presented on Figure S14a.

**Table S20:** ANOVA comparing the dispersion of the replicates for each extraction type and volume at the oceanic site (using Bonferroni corrected *p* values). This table reports the statistical results from the data presented on Figure S14b.

**Table S21:** ANOVA comparing the Shannon diversity indexes of the oceanic site (using Bonferroni corrected *p* values). This table reports the statistical results from the data presented on Figure S16a.

**Table S22:** ANOVA comparing the Shannon diversity indexes of the coastal site (using Bonferroni corrected *p* values). This table reports the statistical results from the data presented on Figure S16b.

**Table S23:** PERMANOVA comparing the taxonomic composition of the reads identified as chloroplasts at the oceanic site (using Bonferroni corrected *p* values). This table reports the statistical results from the data presented on Figure S15a. Data were rarefied to 5,500 reads, then bacteria were removed, and chloroplasts were square root transformed.

**Table S24:** PERMANOVA comparing the taxonomic composition of the reads identified as chloroplasts at the costal site (using Bonferroni corrected *p* values). This table reports the statistical results from the data presented on Figure S15b. Data were rarefied to 5,500 reads, then bacteria were removed, and chloroplasts were square root transformed.

**Table S25:** PERMANOVA comparing the taxonomic composition of the reads identified as chloroplasts at the two sampling sites (using Bonferroni corrected *p* values). This table reports the statistical results from the data presented on Figure S15c. Data were rarefied to 5,500 reads, then bacteria were removed, and chloroplasts were square root transformed.

**Table S26:** ANOVA comparing the dispersion of the replicates for the chloroplast taxonomy from the oceanic site (using Bonferroni corrected *p* values). This table reports the statistical results from the data presented on Figure S15d.

**Table S27:** ANOVA comparing the dispersion of the replicates for the chloroplast taxonomy from the coastal site (using Bonferroni corrected *p* values). This table reports the statistical results from the data presented on Figure S15d.

**Table S28:** ANOVA comparing the dispersion of the replicates from the oceanic site (using Bonferroni corrected *p* values). This table reports the statistical results from the data presented on Figure S10.

**Detailed Protocols**

**List of equipment needed:**

Microcentrifuge

Magnetic rack

UV crosslinker

UV cabinet

**Chemical Lysis: Reagent List:**

1. **Sucrose-Lysis-Buffer:** (0.75 M Sucrose, 40 mM EDTA, 50 mM Tris base, **pH 8.5**):

Calculate required grams needed using the following equation:

(Molarity desired) × (MW of chemical) × (desired volume in Litres)

Calculation to make 50 mL of buffer:

(0.75 M sucrose) × (342.3 g/mol) × (0.05 L) = 12.836 grams needed

(0.04 M EDTA-Na) × (336.21 g/mol) × (0.05 L) = 0.6724 grams needed

(0.05 M Tris base) × (121.14 g/mol) × (0.05 L) = 0.302 grams needed

Adjust Sucrose-Lysis-Buffer solution to pH 8.5 by titrating with a Tris base solution made in DIW (pH ~10). If the pH of this buffer is lower than 8.4, some precipitate may form downstream during the extraction. We suggest making a test extraction and checking the pH prior to extracting valuable samples. If the pH after mixing samples into the buffer is lower than 8.4, we suggest increasing the pH of the Sucrose-Lysis-Buffer incrementally to reach 8.4-8.5.

Aliquot into 1.5 mL crosslinked tubes.

UV for 1 hr on ice in a crosslinker.

Store at 4°C.

1. **Lysozyme (100 mg/ml stock):** weight the correct amount into crosslinked tubes and store dry at -20°C. Resuspend just before use with cross-linked MilliQ
2. **Proteinase K (20 mg/ml stock):** weight the correct amount into crosslinked tubes and store dry at -20°C. Resuspend just before use with cross-linked MilliQ
3. **Sodium dodecyl sulfate (SDS, 25%):** made with cross-linked MilliQ

Calculation to make 50 mL of 25% SDS:

12.5 grams SDS into 50 mL MilliQ; make >24 hrs before intended use as it takes a long time to dissolve.

Aliquot into 1.5 mL crosslinked tubes.

Store at room temperature.

1. **AMPure Beads (Beckman Coulter Inc., USA):** Shake the bottle vigorously prior to pipetting beads.

Store at 4°C.

1. **80% Ethanol in MilliQ:** Crosslink MilliQ beforehand for 1 hr, dilute 100% EtOH to 80% directly prior to use.
2. **Elution Buffer (10 mM Tris-HCl):**

Calculation to make 10 mM Tris:

(0.01 Molar) × (121.14 g/mol) × (0.05 L) = 0.06057 grams needed in 50 mL of crosslinked MilliQ.

Aliquot into 1.5 mL crosslinked tubes.

Crosslink aliquots for 1 hr on ice.

Store at -20°C.

**Chemical Lysis: Extraction Procedure**

The volumes indicated below are for a 100 µL sample, for other inputs see Table S3.

1. Mix 100 µL of samples with 80 µL of Sucrose-Lysis-Buffer

2. Mix by inverting the tube + quick spin

3. Add 30 μL of Lysozyme of (100 mg/mL stock)

4. Mix by inverting the tube + quick spin

5. Incubate at 37°C for 45 min

6. Add 20 μL of SDS (25% solution)

7. Shake and incubate at 70°C for 10 min. DO NOT EXCEED 10 MIN

8. Cool to room temperature, add 8 μL Proteinase K (20 mg/ml stock)

9. Incubate at 37°C for 45 min

10. Mix by inverting the tube + quick spin

11. AMPure beads clean-up

i) Vigorously shake beads bottle and add 476 µL of AMPure beads to the lysed samples (return beads to the fridge during 15 min incubation)

ii) Mix by inverting the tube + quick spin

iii) Incubate for 15 min at room temperature

iv) Place the tube on magnetic stand for 10 min

v) Remove and discard supernatant with pipette, without disturbing beads

vi) Wash 2 times with 80% EtOH (600 µL per tube)

vii) Use a 20 µL pipette to remove residual EtOH

viii) Air dry open tubes on magnetic stand for (15 min)

ix) Remove tubes from magnetic stand

x) Add 20 µL of Elution Buffer (10 mM Tris-HCl)

xi) Mix by pipetting up and down 10 times

xii) Incubate for 2 min at room temperature

xiii) Place on magnetic stand and wait for the liquid to clear (~ 10 min)

xiv) Transfer 18 µL to a new tube

12. Store purified DNA at -20°C (or at -80°C, for no longer than 6 months)

**Physical Lysis: Reagent List:**

1. **DTT-Lysis-Buffer**:

Make KOH stock (0.215 g/10 mL) using crosslinked MilliQ

Make DTT (dithiothreitol) stock (0.2 g/25 mL) using crosslinked MilliQ

Mix the two stocks in the following ratio in crosslinked tubes:

700 µL of KOH + 430 µL of DTT + 520 µl of crosslinked MilliQ

**Ensure that the pH of the solution is equal to 12** (adjust pH by adding more KOH if necessary).

Crosslink aliquots for 1 hr on ice.

Store at -20°C.

1. **Stop Buffer:**

Tris-HCl 4 g/10 mL of crosslinked MilliQ (**pH = 5**)

Aliquot into 1.5 mL crosslinked tubes.

Crosslink aliquots for 1 hr on ice.

Store at -20°C.

1. **AMPure Beads (Beckman Coulter Inc., USA):** Shake the bottle vigorously prior to pipetting beads.

Store at 4°C.

1. **80% Ethanol in MilliQ:** Crosslink MilliQ beforehand for 1 hr, dilute 100% EtOH to 80% directly prior to use.
2. **Elution Buffer (10 mM Tris-HCl):**

Calculation to make 10 mM Tris:

(0.01 Molar) × (121.14 g/mol) × (0.05 L) = 0.06057 grams needed in 50 mL of crosslinked MilliQ.

Aliquot into 1.5 mL crosslinked tubes.

Crosslink aliquots for 1 hr on ice.

Store at -20°C.

**Physical Lysis: Extraction Procedure**

The volumes indicated below are for a 100 µL sample, for other input see Table S2.

1. Do a test to verify that the pH is correct (test with pH strips)

i) Mix a sample + DTT-Lysis-Buffer (see volume below)

ii) Add the Stop Buffer

iii) Test the pH, it should be ~8

2. Mix 100 µl of sample with 75 µl of DTT-Lysis-Buffer

3. Mix by inverting the tube + quick spin

4. Incubate for 10 min at room temperature. DO NOT EXCEED 10 MIN

5. Put in a -80°C freezer for at least 10 min (samples can stay in the freezer for up to 4 hours if samples are processed in batches in subsequent steps)

6. Incubate at 55°C for 5 min (heat block) or until fully thawed. DO NOT EXCEED 5 MIN

7. Add 75 µl of Stop Buffer

8. Mix by inverting the tube + quick spin

9. AMPure beads clean-up

i) Vigorously shake beads bottle and add 500 µL of AMPure beads to the lysed samples (return beads to the fridge during 15 min incubation)

ii) Mix by inverting the tube + quick spin

iii) Incubate for 15 min at room temperature

iv) Place the tube on magnetic stand for 10 min

v) Remove and discard supernatant with pipette

vi) Wash 2 times with 80% EtOH (600 µl per tube)

vii) Use a 20 µl pipette to remove residual EtOH

viii) Air dry open tubes on magnetic stand for 15 min

ix) Remove tubes from magnetic stand

x) Add 20 µl of Elution Buffer (10 mM Tris-HCl)

xi) Mix by pipetting up and down 10 times

xii) Incubate for 2 min at room temperature

xiii) Place on magnetic stand and wait for the liquid to clear (10 min)

xiv) Transfer 18 µl to a new tube

10. Store purified DNA at -20°C (or at -80°C, for no longer than 6 months)
